# Exploring Diagnostic Precision and Triage Proficiency: A Comparative Study of GPT-4 and Bard in Addressing Common Ophthalmic Complaints

**DOI:** 10.3390/bioengineering11020120

**Published:** 2024-01-26

**Authors:** Roya Zandi, Joseph D. Fahey, Michael Drakopoulos, John M. Bryan, Siyuan Dong, Paul J. Bryar, Ann E. Bidwell, R. Chris Bowen, Jeremy A. Lavine, Rukhsana G. Mirza

**Affiliations:** 1 Department of Ophthalmology, Feinberg School of Medicine, Northwestern University, Chicago, IL 60611, USA; 2 Division of Biostatistics, Department of Preventive Medicine, Feinberg School of Medicine, Northwestern University, Chicago, IL 60611, USA

**Keywords:** artificial intelligence, ophthalmology, triage, chatbots, ChatGPT, bard, large language models

## Abstract

In the modern era, patients often resort to the internet for answers to their health-related concerns, and clinics face challenges to providing timely response to patient concerns. This has led to a need to investigate the capabilities of AI chatbots for ophthalmic diagnosis and triage. In this in silico study, 80 simulated patient complaints in ophthalmology with varying urgency levels and clinical descriptors were entered into both ChatGPT and Bard in a systematic 3-step submission process asking chatbots to triage, diagnose, and evaluate urgency. Three ophthalmologists graded chatbot responses. Chatbots were significantly better at ophthalmic triage than diagnosis (90.0% appropriate triage vs. 48.8% correct leading diagnosis; *p* < 0.001), and GPT-4 performed better than Bard for appropriate triage recommendations (96.3% vs. 83.8%; *p* = 0.008), grader satisfaction for patient use (81.3% vs. 55.0%; *p* < 0.001), and lower potential harm rates (6.3% vs. 20.0%; *p* = 0.010). More descriptors improved the accuracy of diagnosis for both GPT-4 and Bard. These results indicate that chatbots may not need to recognize the correct diagnosis to provide appropriate ophthalmic triage, and there is a potential utility of these tools in aiding patients or triage staff; however, they are not a replacement for professional ophthalmic evaluation or advice.

## 1. Introduction

Conversational artificial intelligence (AI) chatbots have gained significant momentum over the last few years. OpenAI’s Chat Generative Pre-trained Transformer (ChatGPT), released November 2022, and Google’s Bard, launched March 2023, are two chatbots that are publicly available. These systems use large language models (LLMs) to process and generate text similar to human language. LLMs constitute a growing field of technology where computer models are pre-trained on large-scale data to then be adapted to a variety of tasks [1]. While there are several LLMs available today, this work will focus its efforts on ChatGPT and Bard due to their widespread presence and public availability. There are a few key operational differences between the two systems. Namely, ChatGPT uses GPT-3.5 or GPT-4 chatbot models, whereas Bard uses PaLM 2 (Pathways Language Model 2). In addition, Bard draws its data live and directly from Google, whereas ChatGPT operates based on data from 2021, and must search papers to gather information [2,3].

These powerful tools are increasingly being considered for efficiency improvements across medicine, including in such applications as supporting clinical practice, scientific writing, image analysis, or immediate medical advice [4,5]. However, they are not without risk. It has been noted that LLMs can produce biased or harmful content due to the vast variability in quality of the data used to power them; in medicine in particular, the quality of chatbot output is of concern as it relates to patient care [6]. There has recently been a strong interest in exploring the capabilities of these LLMs in medicine. Early work demonstrated that OpenAI’s GPT-3.5 performed at or near passing for all three exams in the United States Medical Licensing Exam (USMLE) series [7]. With newer iterations, GPT-4, released March 2023, was found to outperform GPT-3.5 in correctly answering USMLE questions involving communication skills, ethics, empathy, and professionalism [8]. More recent studies have compared GPT-4 and Bard in their performance of answering board-style questions in various subspecialties, and these showed that GPT-4 was superior to Bard [9,10,11]. Within ophthalmology, there has been an interest as well, where in one study GPT-4 demonstrated an excellent performance, significantly better than GPT-3.5, in answering practice questions to the Ophthalmology Knowledge Assessment Program (OKAP) examination [12].

As AI chatbots evolve, become more widely used by the general population, and are integrated into common internet search engines, it is increasingly imperative to assess their role in the patient care journey. It is a well-established trend that patients look to the internet for seeking information about their health [13,14] and often turn to the internet first for health advice before contacting health professionals [15,16]. Moreover, patients often have long wait times when they do contact their health providers, which is especially true in ophthalmology. A recent study projected that there will be a sizable shortage of ophthalmologists relative to demand by the year 2035 [17], with limited ophthalmology coverage in emergency departments, especially in rural settings [18]. As a result, non-ophthalmology providers, busy triage call centers, and patients may begin to look to technological solutions such as AI chatbots that can support addressing ophthalmic complaints and triage. It is therefore critical that the strengths and potential risks of these tools are evaluated thoroughly.

Recent studies have begun to explore the capabilities of AI chatbots as ocular symptom checkers or ophthalmic triage tools. Specifically, Pushpanathan et al. investigated accuracy and quality of responses (without examining triage capabilities) for GPT-3.5, GPT-4, and Bard in answering direct questions about specific ocular symptoms and found that GPT-4 had the highest accuracy [19]. Lim et al. benchmarked performance of ChatGPT and Bard for myopia-related queries specifically, and also found that GPT-4.0 had superior accuracy [20]. Lyons et al. compared the triage capabilities of GPT-4, Bing Chat, and WebMD Symptom Checker with ophthalmology trainees across 24 ophthalmic diagnoses. Notably, GPT-4 performed comparably with the trainees in diagnostic and triage accuracy [21].

In this work, we aimed to evaluate and compare GPT-4 and Bard in their responses to commonly encountered ophthalmic complaints corresponding to 40 critical diagnoses in the form of simulated patient vignettes with targeted questions about proposed diagnoses and triage recommendations. We additionally analyzed how prompt descriptiveness impacts response quality with the aim to better understand how they would best be used in future patient-oriented settings. As we gain a better understanding of the values and limitations of this technology, we can move closer to determining how conversational AI can potentially be implemented in day-to-day society for meeting the demands of delivering timely, accurate, and safe ophthalmic health information for patient use and decision making.

## 2. Materials and Methods

The Northwestern University Institutional Review Board determined that this in silico research did not involve human subjects. At the time of data collection, GPT-4 was publicly available by paid subscription through ChatGPT Plus, and Bard was freely accessible.

### 2.1. Creation of Simulated Patient Prompts in Ophthalmology

**Scheme 1:**
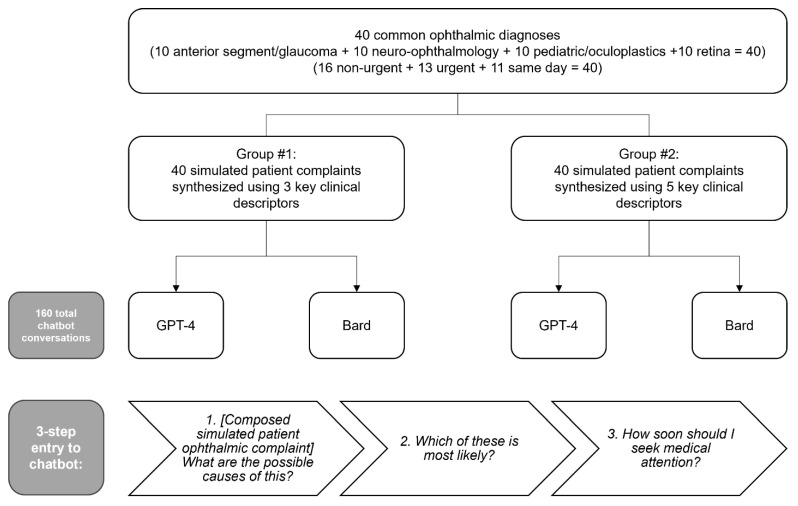
Flowchart of overall study design of chatbot prompts.

We systematically constructed common scenarios encountered in ophthalmology from the perspective of a patient. Forty common diagnoses, including “cannot miss diagnoses” [22], were identified and distributed evenly among four groups of ophthalmic specialties: anterior segment/glaucoma, neuro-ophthalmology, pediatric ophthalmology/oculoplastics, and retina. An urgency level to seek care was designated for each diagnosis as either same day, urgent (<1 week), or non-urgent (>1 week). For each diagnosis, two prompts were created, one with three key clinical descriptors and one with five descriptors (Scheme 1). A descriptor was defined as a clinically relevant piece of information that addressed any of the following: relevant history, onset, duration, laterality, mention of specific ocular anatomy, vision, dyschromatopsia, pain, photophobia, visual disturbances, or any other clinical characteristic. For each patient scenario, consensus was reached among experienced ophthalmologists (P.B., A.E.B., and R.C.B) regarding both intended diagnosis and urgency level based on expert opinion (Supplemental Tables S1–S4).

### 2.2. Input to Artificial Intelligence Chatbots

The simulated patient prompts were entered into the AI chatbots between 14 June 2023 and 20 June 2023 using GPT-4 version 2023.05.24 and Bard version 2023.06.07. While many chatbots exist, including Microsoft Bing AI, Claude AI, or Meta’s LLaMA, we chose these two as they are among the most commonly used and referenced chatbots at the time of publication, and they are publicly available. Each prompt was entered into the chatbot using a standardized 3-part stepwise approach. First, the simulated patient scenario followed by the question “What are the possible causes of this?” was entered to the chatbot. The second entry was “Which of these is most likely?”. Finally, the third input was “How soon should I seek medical attention?” Chatbot history was reset prior to starting each 3-part entry. These sequential questions were to ensure that the chatbot addressed a differential diagnosis, leading diagnosis, and provided triage recommendations.

### 2.3. Grading of Chatbot Responses

To evaluate the response generated by the chatbots, a seven-question questionnaire was designed using both a 4-point Likert scale and binary (yes/no) style questions (Supplemental Table S5). Prior to grading, chatbot identifiers (e.g., “I’m an AI developed by OpenAI”) were removed from chatbot responses to eliminate potential grader bias toward a particular chatbot. Two experienced ophthalmologists graded each chatbot conversation for accuracy of diagnosis and appropriateness of triage recommendations (primary outcomes), as well as relevance of differential diagnosis, satisfaction with quality of responses for real patient use, and potential harm that responses may pose to real patients (secondary outcomes). A third experienced ophthalmologist served as arbiter for any grading disagreements. All graders were blinded to the chatbot source. For the purposes of this analysis, responses of 3 or 4 on the 4-point Likert scale were treated as “agree” and responses of 1 or 2 were treated as “disagree” to provide binary data.

### 2.4. Statistical Analysis

Descriptive statistics were generated for all variables of interest where frequencies along with percentages were reported. To compare the outcomes of interest between ChatGPT and Bard, as well as between 5 and 3 descriptors, Pearson’s Chi-squared test or Fisher’s exact test was used when appropriate. Sub-analyses for sub-specialty categories and urgency levels were conducted using the same method. Logistic regression models were applied to the primary outcomes with the degree of detail in prompt as the predictor, and the models were fit separately for ChatGPT and Bard. Model performance was estimated using Area under Curve (AUC) and receiver operating characteristic (ROC) curves. All analyses were conducted using R version 4.3.1.

## 3. Results

Eighty unique entries were supplied to both GPT-4 and Bard, resulting in a total of 160 chatbot generated responses. 40 entries had 3 prompt descriptors and a counterpart 40 entries had 5 prompt descriptors. The 40 diagnoses were broken down into 4 sub-specialty categories (10 general, 10 neuro-ophthalmology, 10 pediatrics/oculoplastics, and 10 retina) and 3 urgency levels (16 non-urgent, 13 urgent, and 11 same day) (Scheme 1). The diagnosis rates (i.e., providing the correct diagnosis as the stated most likely cause of the patient’s symptoms) for GPT-4 and Bard were 53.8% and 43.8%, respectively (*p* = 0.2). Interestingly, both chatbots were significantly better at providing triage recommendations than at providing the correct leading diagnosis (GPT-4: *p* < 0.001, Bard: *p* < 0.001). The rates of generally appropriate triage recommendations for GPT-4 and Bard were 96.3% and 83.8%, respectively (*p* = 0.008) (Table 1).

Secondary outcomes included relevance of differential diagnosis, grader satisfaction with chatbot responses, and expert opinion as to whether the chatbot response could pose harm if provided to an actual patient. Of all the 160 responses, the differential diagnoses were generally relevant (87.5%). Additionally, graders indicated satisfaction with 109 responses (68.1%); the satisfaction rate was significantly higher for responses from GPT-4 than from Bard (81.3% vs. 55.0%, respectively; *p* < 0.001). Graders reported that 21 of 160 chatbot responses (13.1%) would pose harm if provided to an actual patient; GPT-4 had a lower potential harm rate than Bard (6.3% vs. 20.0%; *p* = 0.010) (Table 1).

Additional sub-analyses were performed comparing the 3 and 5 descriptor cohorts. Notably, increasing the degree of prompt descriptiveness resulted in significant improvement in diagnosis rates for both GPT-4 and Bard (GPT-4: 42.5% vs. 65.0%, respectively; *p* = 0.044, Bard: 32.5% vs. 55.0%, respectively; *p* = 0.043), whereas triage recommendations did not significantly improve (Table 2). However, the model performance of prompt descriptiveness predicting appropriate triage for ChatGPT (AUC 0.587) was better than Bard (AUC 0.523) (Supplemental Figure S1). Of note, increasing the number of descriptors (from 3 to 5) resulted in significantly higher grader satisfaction for GPT-4 (70.0% vs. 92.5%; *p* = 0.010), but not for Bard (47.5% vs. 62.5%; *p* = 0.2) (Table 2).

Further sub-analyses were performed to compare GPT-4 with Bard and this demonstrated that GPT-4 performed better than Bard in the 5 descriptor cohort when considering the responses that listed the correct diagnosis anywhere within the chatbot response (92.5% vs. 72.5%; *p* = 0.019). GPT-4 also performed significantly better than Bard in the 5 descriptor cohort in generating relevant differential diagnoses (95.0% vs. 77.5%; *p* = 0.023). The satisfaction rate was also significantly higher for GPT-4 than Bard (3 descriptor group: 70.0% vs. 47.5%; *p* = 0.041, 5 descriptor group: 92.5% vs. 62.5%; *p* = 0.001). Within the 5 descriptor cohort, the rate of potential to cause patient harm was zero for GPT-4 and 15.0% for Bard (*p* = 0.026) (Table 3).

Additional sub-analyses of the GPT-4–5 descriptor cohort revealed that the chatbot performed similarly for all outcome measures regardless of urgency level and subspecialty of diagnosis (Supplemental Tables S6 and S7).

## 4. Discussion

To our knowledge, this is the first work to investigate both the diagnostic accuracy and appropriateness of triage recommendations of GPT-4 and Bard in response to simulated ophthalmic complaints of varying degrees of descriptiveness. It also uses the largest sample size of responses. Overall, the chatbots were significantly better at ophthalmic triage than at providing the correct diagnosis; notably, GPT-4 displayed high rates of appropriate triage—which supports data found in another recent study [21]. Our work demonstrates that GPT-4 performed significantly better than Bard in the domains of appropriate triage recommendations, responses that experts were satisfied with for patient use, and responses that were not considered to cause harm if given to real patients. While some of the results were not statistically significant in the sub-analyses, this was likely due to the smaller sample size. It should be highlighted, however, that in the 5 descriptor sub-analysis, GPT-4 performed significantly better than Bard in considering the correct diagnosis as either the most likely diagnosis or as one of the possible diagnoses in the differential diagnosis (92.5% vs. 72.5%; *p* = 0.019). This is in agreement with recent research demonstrating the superiority of GPT-4 to Bard in correctly answering questions related to ocular symptoms [19] and myopia-related queries [20]. In addition, our work uniquely reveals that increasing the detail of chatbot input (more descriptors) generally improved the quality of output. It should be emphasized that the chatbots were able to provide appropriate triage recommendations without necessarily recognizing the exact diagnosis which better lends itself as an ophthalmic triage tool than as a diagnostic tool.

Another critical question to consider is the performance of these chatbots in the context of do-not-miss diagnoses that are vision- or life-threatening, such as an oculomotor nerve palsy, endophthalmitis, or acute angle closure crisis. In these cases, humans might be trained to take extreme caution when giving guidance to patients, and adoption of conversational AI tools in this space may depend on the responses in such cases. Here, we examined the superior performing chatbot (GPT-4) under optimal conditions (5 descriptor prompts), and we found that all 11 entries with do-not-miss diagnoses resulted in generally appropriate triage recommendations and responses that senior ophthalmologists were satisfied with for patient use. Moreover, there were no responses (0 of 40) in the GPT-4–5 descriptor subgroup that were considered to be potentially harmful. This is particularly valuable as we consider the potential applications of this technology for future patient use, either as a self-inquiry tool or as an adjunct tool for medical staff to execute timely, appropriate, and safe patient triage.

This study’s moderate sample size of chatbot responses is one of its many strengths. We used a highly systematic approach to develop and input all chatbot entries, with chatbot history being reset following each entry to eliminate the variable of chatbot growth over the course of data collection. In addition, our 3-step approach to inputting entries for each “conversation” attempted to take advantage of the conversational capabilities of these chatbots. Lastly, the chatbot responses were all gathered within a one-week time-frame during which all responses were generated from a single iteration of either GPT-4 or Bard, then statistical analysis was performed.

Based on the results found in this work, chatbot responses in the current state of technology are promising but not a sufficient substitute for professional medical advice, yet only in a handful of chatbot responses, GPT-4 more often than Bard, were there such explicit disclaimers. Some examples from GPT-4 include: “I’m an AI developed by OpenAI and while I can help suggest some potential causes for your symptoms, I’m not a substitute for professional medical advice”, “Please note that this advice does not substitute professional medical advice. Always consult with a healthcare provider for medical concerns”, or “Remember that while the internet can provide useful general advice, it’s no substitute for the professional judgment of a healthcare provider who can evaluate your child in person”. It should also be added that in less than half of the GPT-4 responses (28 of 80) and in only one Bard response, was there a specific comment about being an AI; interestingly, Bard only indicated itself as such when unable to respond [“I’m a text-based AI and can’t assist with that.”]. In this work, we inputted the chatbot prompts from the perspective of a simulated patient; in the future, it would be worthwhile to assess how chatbot responses would differ if the chatbot was prompted to answer questions while identifying itself as an AI-generated ophthalmic triage staff. Nonetheless, the chatbot’s recognition of self-limitations as an AI and its recommendation to seek professional medical evaluation are important elements that serve as safety checks to the general public who may already be using this technology to answer their own health-related questions.

## 5. Limitations

Prior to considering the implementation of such technology for clinical use, further studies with larger data sets should be performed. Another shortcoming of this study is the relatively subjective definition of a “descriptor”, where some modifiers might be more informative than others; nonetheless, our results overall do show that a more descriptive chatbot prompt is desirable. A general concern of conversational AI that must also be highlighted is the risk of generating “hallucinations”, seemingly accurate information that are in fact false [23]. While our study found that chatbot responses, especially GPT-4, were typically not harmful, we did not specifically investigate the number of hallucinatory responses. The potential of generating convincing misinformation is a serious concern that should not be taken lightly and should be further explored, especially as different iterations of these software are developed.

## 6. Conclusions

In this work we have evaluated two AI chatbots for their use in ophthalmology; however, as the usage of these tools increases over the coming years, it is imperative to continue their evaluation. As future iterations of GPT-4, Bard, or other LLMs are published, each of these should be tested anew. Additionally, more reviewers could assess a larger sample size of chatbot responses in order to provide greater accountability for the variability in AI responses. In addition, the language of prompts can be more varied in future studies to allow for variability from the user. Finally, more in-depth studies of chatbots’ use may be performed over a longitudinal study following patients’ real-time diagnoses and the chatbots’ capabilities in diagnosis and triage.

Another aspect of AI chatbots to study further includes incorporating images with written text of patient concerns to assess how diagnostic accuracy and triage recommendations vary with the added variable of clinical photos. Given that ophthalmology is a highly visual discipline, it would be interesting to assess how external photos of the eye (i.e., the type of photo that a patient could realistically provide) in conjunction with clinical context would impact chatbot responses.

While this work sheds light on the performance and potential utility of GPT-4 and Bard in the domain of ophthalmic diagnostics and triage, the broader scientific understanding of conversational AI in medicine is still in its infancy as there is an endless number of ways to engage with these chatbots. Recognizing the optimal approach of feeding information into the chatbot and evaluating the quality of the resultant response is imperative to advancing towards real-world application of conversational AI for patient use, either as a self-triage tool or as an adjunct triage tool for medical staff. Our results herein suggest that currently, GPT-4 outperforms Bard and that a greater number of key clinical descriptors for chatbot input is desirable. But only until we have established a full understanding of the strengths and weaknesses of AI chatbots and have been able to consistently achieve a high level of excellence in the quality of responses should we consider their incorporation in patient care. As the world quickly moves towards greater use of conversational AI and a greater need in clinical settings for technological solutions, the urgency for investigative studies like this one will only increase.

## Figures and Tables

**Table 1:** Primary and secondary outcomes of Bard and GPT-4.

	Overall N = 160 ^1^	Bard N = 80 ^1^	GPT-4 N = 80 ^1^	*p*-Value ^2^
Primary outcomes of Bard and GPT-4 overall				
Correct diagnosis as most likely cause of symptoms	78 (48.75%)	35 (43.75%)	43 (53.75%)	0.2
Correct diagnosis somewhere in the chatbot conversation	125 (78.13%)	58 (72.50%)	67 (83.75%)	0.085
“Somewhat” or “completely” appropriate triage recommendations	144 (90.00%)	67 (83.75%)	77 (96.25%)	0.008 *
“Completely” appropriate triage recommendations	123 (76.88%)	55 (68.75%)	68 (85.00%)	0.015 *
Secondary outcomes of Bard and GPT-4 overall				
“Somewhat” or “very” relevant differential diagnosis	140 (87.50%)	66 (82.50%)	74 (92.50%)	0.056
Graders “somewhat” or “very” satisfied with quality of chatbot response for actual patient use	109 (68.13%)	44 (55.00%)	65 (81.25%)	<0.001 *
Potentially harmful for patients	21 (13.13%)	16 (20.00%)	5 (6.25%)	0.010 *

* *p*-value < 0.05. ^1^ N (%). ^2^ Pearson’s Chi-squared test between Bard and GPT-4.

**Table 2:** Primary and secondary outcomes between 3 and 5 descriptors for Bard and GPT-4.

	Bard			GPT-4		
	3 Descriptors N = 40 ^1^	5 Descriptors N = 40 ^1^	*p*-Value ^2^	3 Descriptors N = 40 ^1^	5 Descriptors N = 40 ^1^	*p*-Value ^3^
Primary outcomes of Bard and GPT-4 overall						
Correct diagnosis as most likely cause of symptoms	13 (32.50%)	22 (55.00%)	0.043 *	17 (42.50%)	26 (65.00%)	0.044 *
Correct diagnosis somewhere in the chatbot conversation	29 (72.50%)	29 (72.50%)	>0.9	30 (75.00%)	37 (92.50%)	0.034 *
“Somewhat” or “completely” appropriate triage recommendations	33 (82.50%)	34 (85.00%)	0.8	38 (95.00%)	39 (97.50%)	>0.9
“Completely” appropriate triage recommendations	24 (60.00%)	31 (77.50%)	0.091	32 (80.00%)	36 (90.00%)	0.2
Secondary outcomes of Bard and GPT-4 overall						
“Somewhat” or “very” relevant differential diagnosis	35 (87.50%)	31 (77.50%)	0.2	36 (90.00%)	38 (95.00%)	0.7
Graders “somewhat” or “very” satisfied with quality of chatbot response for actual patient use	19 (47.50%)	25 (62.50%)	0.2	28 (70.00%)	37 (92.50%)	0.010 *
Potentially harmful for patients	10 (25.00%)	6 (15.00%)	0.3	5 (12.50%)	0 (0.00%)	0.055

* p-value < 0.05. ^1^ N (%). ^2^ Pearson’s Chi-squared test. ^3^ Fisher’s exact test; Pearson’s Chi-squared test.

**Table 3:** Primary and secondary outcomes between Bard and GPT-4 for 3 and 5 descriptor cohorts.

	3 Descriptors			5 Descriptors		
	Bard N = 40 ^1^	GPT-4 N = 40 ^1^	*p*-Value ^2^	Bard N = 40 ^1^	GPT-4 N = 40 ^1^	*p*-Value ^2^
Primary outcomes of Bard and GPT-4 overall						
Correct diagnosis as most likely cause of symptoms	13 (32.50%)	17 (42.50%)	0.4	22 (55.00%)	26 (65.00%)	0.4
Correct diagnosis somewhere in the chatbot conversation	29 (72.50%)	30 (75.00%)	0.8	29 (72.50%)	37 (92.50%)	0.019 *
“Somewhat” or “completely” appropriate triage recommendations	33 (82.50%)	38 (95.00%)	0.2	34 (85.00%)	39 (97.50%)	0.11
“Completely” appropriate triage recommendations	24 (60.00%)	32 (80.00%)	0.051	31 (77.50%)	36 90.00%)	0.13
Secondary outcomes of Bard and GPT-4 overall						
“Somewhat” or “very” relevant differential diagnosis	35 (87.50%)	36 (90.00%)	>0.9	31 (77.50%)	38 (95.00%)	0.023 *
Graders “somewhat” or “very” satisfied with quality of chatbot response for actual patient use	19 (47.50%)	28 (70.00%)	0.041 *	25 (62.50%)	37 92.50%)	0.001 *
Potentially harmful for patients	10 25.00%)	5 (12.50%)	0.2	6 (15.00%)	0 (0.00%)	0.026 *

* *p*-value < 0.05. ^1^ N (%). ^2^ Fisher’s exact test; Pearson’s Chi-squared test.

## Data Availability

The original contributions presented in the study are included in the article/supplementary material, further inquiries can be directed to the corresponding author.

## Supplementary Materials

The following supporting information can be downloaded at: https://www.mdpi.com/article/10.3390/bioengineering11020120/s1, Table S1: Compilation of anterior segment/glaucoma/comprehensive simulated patient complaints; Table S2: Compilation of neuro-ophthalmology simulated patient complaints; Table S3: Compilation of pediatric/oculoplastic simulated patient complaints; Table S4: Compilation of retina simulated patient complaints; Table S5: Questionnaire to grade chatbot responses; Table S6: Sub-analysis of primary and secondary outcomes of GPT-4–5 descriptor cohort by urgency level; Table S7: Sub-analysis of primary and secondary outcomes of GPT-4–5 descriptor cohort by subspecialty; Figure S1: Receiver operating characteristic curves for Bard and GPT-4 in providing triage recommendations.
