# Supplementary material for: Exploring Diagnostic Precision and Triage Proficiency: A Comparative Study of GPT-4 and Bard in Addressing Common Ophthalmic Complaints
Source: Bioengineering (Basel). 2024 Jan 26;11(2):120. doi: 10.3390/bioengineering11020120 (PMC10886029; doi:10.3390/bioengineering11020120)
Supplement: Supplementary file 1 [file bioengineering-11-00120-s001.zip › bioengineering-2834802-supplementary.pdf]

Table S1. Compilation of anterior segment/glaucoma/comprehensive simulated patient complaints.

**Anterior segment/glaucoma/ comprehensive**			
**Pre-specified simulated ophthalmologic diagnosis**	**Pre-specified acuity**	**3 descriptor simulated patient ophthalmic complaint**	**5 descriptor simulated patient ophthalmic complaint**
Acute angle closure glaucoma	Same Day	My eye is painful, and I see a lot of halos.	I had a dilated eye exam earlier today and now my eye is painful and I am noticing a lot of halos.
Corneal ulcer	Same Day	My eye is very painful and red.	I fell asleep with my contact lenses and now my eye is very painful and red.
Chemical injury	Same Day	My right eye is painful and tearing.	I accidentally splashed some bleach in my right eye and now it is painful and tearing.
Recurrent corneal erosion	Non-urgent	I had pain in my right eye that resolved in a few hours.	I woke up with pain in my right eye that resolved in a few hours. This has happened many times in the past.
Anterior uveitis	Urgent	I have right eye pain and extreme light sensitivity.	I have new right eye pain, extreme light sensitivity, and my joints have been hurting more than usual.
Traumatic iritis	Urgent	My right eye is light sensitive and a little blurred.	A ball hit me in the right eye a few days ago and I’ve been light sensitive and a little blurred.
Acute rise in intraocular pressure/pigment dispersion syndrome	Non-urgent	I have blurred vision and glare after strenuous exercise.	I have been noticing blurred vision in both eyes and glare after strenuous exercise.
Subconjunctival hemorrhage	Non-urgent	There is a red spot on my eye – it looks like it’s bleeding.	I was coughing hard all day today and now there is a red spot on

			my eye – it looks like it’s bleeding.
Posterior capsular opacification	Non-urgent	The vision in the eye I had cataract surgery 1 year ago is blurry.	I had cataract surgery one year ago and my vision was pretty good after that. Now the vision in that eye is blurry again.
Glaucoma suspect / ocular hypertension	Non-urgent	My eye pressure was a little higher than normal in both eyes. My vision is fine.	I was at an eye screening event, and they told me my eye pressure was a little higher than normal in both eyes. My vision is fine and I don’t feel anything wrong.

Table S2. Compilation of neuro-ophthalmology simulated patient complaints.

Neuro-ophthalmology			
**Pre-specified simulated ophthalmologic diagnosis**	**Pre-specified acuity**	**3 descriptor simulated patient ophthalmic complaint**	**5 descriptor simulated patient ophthalmic complaint**
Horner syndrome with carotid dissection	Same Day	My neck is very painful, and my eye is a little droopy.	I just had a session with my chiropractor and now my neck is very painful, and my eye is a little droopy.
Giant cell arteritis	Same Day	I just lost vision in my left eye.	I have been losing weight, have been experiencing scalp tenderness, and now I lost vision in my left eye.
Occipital Stroke	Same Day	I'm unable to read or see things on the right side of my vision. It seems like both eyes are affected.	I woke up this morning and I'm unable to read or see things on the right side of my vision. It seems like both eyes are affected. Everything was fine yesterday.
Optic neuritis	Urgent	The vision in my right eye appears washed out and I have some pain.	The vision in my right eye has seemed washed out over the last few days. I'm also having some pain with eye movements in that eye.
Ocular Myasthenia gravis	Urgent	My right eyelid droops and gets worse as the day goes on.	I have been noticing my right eyelid drooping. It gets worse as the day goes on and I also notice double vision.

Trochlear nerve palsy	Urgent	I have double vision all the time. The double vision goes away when I close an eye.	I recently hit my head on a cabinet and now I'm noticing double vision all the time. The double vision goes away when I close an eye.
Oculomotor nerve palsy	Same Day	My right eye is suddenly really droopy.	My right eye is suddenly really droopy and it seems like my eyes don't work together. I'm having double vision as well.
Migraine with aura	Non-urgent	I saw a shimmering static light for about 15 minutes and then had a headache.	I saw a shimmering static light for about 15 minutes and then had a headache. I'm 30 years old and I've never had this before.
Orbicularis myokymia	Non-urgent	My right eyelid has been twitching.	My right eyelid has been twitching off and on for several days.
Optic disk drusen	Non-urgent	My optic nerve has a unique appearance to it and there are "little deposits".	My eye doctor recently told me my right optic nerve has a unique appearance to it and that there are "little deposits".

Table S3. Compilation of pediatric/oculoplastic simulated patient complaints.

**Pediatric/oculoplastic**			
**Pre-specified simulated ophthalmologic diagnosis**	**Pre-specified acuity**	**3 descriptor simulated patient ophthalmic complaint**	**5 descriptor simulated patient ophthalmic complaint**
Leukocoria / rule out retinoblastoma	Urgent	I took a picture and in the photo one pupil was very white.	I took a picture of my infant and in the photo one pupil was very white and one pupil looked normal.
Muscle entrapment / Orbital fracture	Same Day	My 12-year-old son has double vision and nausea when he tries to look around.	My 12-year-old son fell on his face and now has double vision and nausea when he tries to look around.
Pre-septal cellulitis	Urgent	My child's eye is swollen.	My toddler had a bug bite near her eye a few days ago and now it's swollen.
Thyroid eye disease	Non-urgent	My eyes bulge out of my face and they feel irritated.	My eyes have recently started to bulge out of my face, and they feel irritated and sensitive.
Strabismus	Non-urgent	My 3-year-old's eyes cross.	My 3-year-old's eyes sometimes cross. She doesn't seem to be able to control it.
Basal cell carcinoma/squamous cell carcinoma	Urgent	I have a bump on my right eyelid that is growing.	I have a bump on my right eyelid that is growing and sometimes bleeds. It's also making me lose my eyelashes.
Dacryocystitis	Urgent	My toddler has a bump by her eye.	My toddler has a painful bump under the corner of her right eye by her nose.
Nasolacrimal duct obstruction	Non-urgent	My 6-month-old's right eye tears up.	My 6-month-old's right eye always tears up and has crusty lashes.

Aponeurotic ptosis	Non-urgent	My right eyelid has been droopy.	I had cataract surgery and my right eyelid has been droopy ever since. It hasn’t gotten better or worse.
Blepharitis	Non-urgent	My eyes itch, and I get flakes in my lashes.	My eyes itch and I get flakes in my lashes, but my vision is good. This has been going on for months.

Table S4. Compilation of retina simulated patient complaints.

Retina			
**Pre-specified simulated ophthalmologic diagnosis**	**Pre-specified acuity**	**3 descriptor simulated patient ophthalmic complaint**	**5 descriptor simulated patient ophthalmic complaint**
Endophthalmitis	Same Day	My eye is very painful, and my vision is very blurry.	I recently had an eyeball injection, and now my eye is very painful and my vision is very blurry.
Retinal detachment	Same Day	I see dots, bright lights, and noticed a shade coming into my vision.	In the last day I started seeing dots and bright lights in my right eye. I also noticed a shade coming into my vision.
Amaurosis fugax	Same Day	The vision became really dark but then improved over about 30 minutes and my vision returned to normal.	The vision in my right eye became really dark, but then it gradually improved over about 30 minutes and my vision returned to normal.
Choroidal neovascular membrane or wet AMD	Urgent	Straight lines appear wavy and things are more blurry in my right eye.	I have macular degeneration and have recently noticed that straight lines have become wavy and things are more blurry in my right eye.
Vitreous hemorrhage/proliferative diabetic retinopathy	Urgent	I just started seeing a lot of red dots in my right eye.	I have diabetes and just started seeing a lot of red dots in my right eye. It is making it difficult to see through.
Acute posterior vitreous detachment	Urgent	I see flashing lights in my vision and a new cobweb-like thing in my right eye.	I just started noticing flashing lights in my vision and see a new cobweb-like thing in my right eye. I keep

			thinking it's a fruit fly.
Central Serous Chorioretinopathy	Urgent	I have a new smudge in the center of my right vision.	I have a new smudge and distortion in the center of my right vision. I have also been very stressed at work.
Gas bubble	Non-urgent	I see a horizontal line halfway in my vision that bobs with my head.	I had a retinal detachment surgery a few weeks ago. I'm now noticing a horizontal line halfway in my vision that bobs with my head.
Floaters	Non-urgent	I sometimes see a floater in my right eye.	I had an episode of flashes and floaters in my right eye years ago that has resolved. I sometimes still see a floater.
Post-injection irritation	Non-urgent	I had an injection in my eye, and it feels like something is in my eye.	I had an injection in my eye today and my eye has felt scratchy. It feels like something is in my eye.

Table S5. Questionnaire to grade chatbot responses.

**Does the chatbot provide the correct diagnosis as the stated most likely cause of the patient's symptoms?**
Yes<br>No<br>N/A, the chatbot does not provide a most likely cause
**Does the chatbot provide the correct diagnosis somewhere in the chatbot conversation, i.e., in the possible causes or as the most likely cause?**
Yes<br>No
**How relevant is the differential diagnosis provided by the chatbot?**
4 – very relevant<br>3 – somewhat relevant<br>2 – somewhat irrelevant<br>1 – very irrelevant
**The chatbot provides an appropriate triage recommendation with respect to timeframe in which to seek medical attention and urgency of issue.**
4 – I completely agree<br>3 – I somewhat agree<br>2 – I somewhat disagree<br>1 – I completely disagree
**If you did NOT completely agree (4) with the triage recommendation provided by the chatbot, do you feel that the chatbot response was an over-triage (i.e., recommending seeking evaluation sooner than indicated or overdoing the severity/urgency of an issue) or an under-triage (i.e., not recommending evaluation soon enough or not emphasizing the severity/urgency of an issue enough)?**

Over-triage<br>Under-triage<br>N/A, I completely agreed (4) with the triage recommendations
**How satisfied would you be if these chatbot responses were given to a real patient?**
4 – very satisfied<br>3 – somewhat satisfied<br>2 – somewhat dissatisfied<br>1 – very dissatisfied
**In your expert opinion, would the chatbot's response pose harm if provided to an actual patient?**
Yes<br>No

Table S6. Sub-analysis of primary and secondary outcomes of GPT-4 – 5 descriptor cohort by urgency level.

**Variable**	**Overall,** N = 40[1]	**Non-Urgent,** N = 16[1]	**Urgent,** N = 13[1]	**Same day,** N = 11[1]	**p-value**[2]
**Does the chatbot provide the correct diagnosis as the stated most likely cause of the patient's symptoms?**					>0.9
No	14 (35.00%)	5 (31.25%)	5 (38.46%)	4 (36.36%)	
Yes	26 (65.00%)	11 (68.75%)	8 (61.54%)	7 (63.64%)	
**Does the chatbot provide the correct diagnosis somewhere in the chatbot conversation, i.e., in the possible causes or as the most likely cause?**					0.10
No	3 (7.50%)	3 (18.75%)	0 (0.00%)	0 (0.00%)	
Yes	37 (92.50%)	13 (81.25%)	13 (100.00%)	11 (100.00%)	
**The chatbot provides an appropriate triage recommendation with respect to timeframe in which to seek medical attention and urgency of issue.**					>0.9
1 or 2 - I completely or somewhat disagree	1 (2.50%)	1 (6.25%)	0 (0.00%)	0 (0.00%)	
3 or 4 - I completely or somewhat agree	39 (97.50%)	15 (93.75%)	13 (100.00%)	11 (100.00%)	
**Do you completely agree with the triage recommendation provided by the chatbot?**					>0.9
I do not completely agree	4 (10.00%)	2 (12.50%)	1 (7.69%)	1 (9.09%)	
I completely agree	36 (90.00%)	14 (87.50%)	12 (92.31%)	10 (90.91%)	

Variable	Overall, N = 40[1]	Non-Urgent, N = 16[1]	Urgent, N = 13[1]	Same day, N = 11[1]	p-value[2]
**How relevant is the differential diagnosis provided by the chatbot?**					0.3
1 or 2 - very or somewhat irrelevant	2 (5.00%)	2 (12.50%)	0 (0.00%)	0 (0.00%)	
3 or 4 - somewhat or very relevant	38 (95.00%)	14 (87.50%)	13 (100.00%)	11 (100.00%)	
**How satisfied would you be if these chatbot responses were given to a real patient?**					0.8
1 or 2 - very or somewhat dissatisfied	3 (7.50%)	2 (12.50%)	1 (7.69%)	0 (0.00%)	
3 or 4 - very or somewhat satisfied	37 (92.50%)	14 (87.50%)	12 (92.31%)	11 (100.00%)	
**In your expert opinion, would the chatbot's response pose harm if provided to an actual patient?**					
No	40 (100.00%)	16 (100.00%)	13 (100.00%)	11 (100.00%)	

[1] n (%)

[2] Fisher's exact test

Table S7. Sub-analysis of primary and secondary outcomes of GPT-4 – 5 descriptor cohort by subspecialty.

**Variable**	**Overall**, N = 40[1]	**Ant. Segment/ Glaucoma**, N = 10[1]	**Neuro-ophth**, N = 10[1]	**Ped/ Oculo -plastics**, N = 10[1]	**Retina**, N = 10[1]	**p-value**[2]
**Does the chatbot provide the correct diagnosis as the stated most likely cause of the patient's symptoms?**						0.7
No	14 (35.00%)	3 (30.00%)	2 (20.00%)	4 (40.00%)	5 (50.00%)	
Yes	26 (65.00%)	7 (70.00%)	8 (80.00%)	6 (60.00%)	5 (50.00%)	
**Does the chatbot provide the correct diagnosis somewhere in the chatbot conversation, i.e., in the possible causes or as the most likely cause?**						0.6
No	3 (7.50%)	2 (20.00%)	0 (0.00%)	0 (0.00%)	1 (10.00%)	
Yes	37 (92.50%)	8 (80.00%)	10 (100.00%)	10 (100.00%)	9 (90.00%)	
**The chatbot provides an appropriate triage recommendation with respect to timeframe in which to seek medical attention and urgency of issue.**						>0.9
1 or 2 - I completely or somewhat disagree	1 (2.50%)	0 (0.00%)	0 (0.00%)	0 (0.00%)	1 (10.00%)	
3 or 4 - I completely or somewhat agree	39 (97.50%)	10 (100.00%)	10 (100.00%)	10 (100.00%)	9 (90.00%)	

Variable	Overall, N = 40[1]	Ant. Segment/ Glaucoma, N = 10[1]	Neuro-ophth, N = 10[1]	Ped/ Oculo -plastics, N = 10[1]	Retina, N = 10[1]	p-value[2]
**Do you completely agree with the triage recommendation provided by the chatbot?**						0.2
I do not completely agree	4 (10.00%)	0 (0.00%)	0 (0.00%)	1 (10.00%)	3 (30.00%)	
I completely agree	36 (90.00%)	10 (100.00%)	10 (100.00%)	9 (90.00%)	7 (70.00%)	
**How relevant is the differential diagnosis provided by the chatbot?**						0.2
1 or 2 - very or somewhat irrelevant	2 (5.00%)	2 (20.00%)	0 (0.00%)	0 (0.00%)	0 (0.00%)	
3 or 4 - somewhat or very relevant	38 (95.00%)	8 (80.00%)	10 (100.00%)	10 (100.00%)	10 (100.00%)	
**How satisfied would you be if these chatbot responses were given to a real patient?**						>0.9
1 or 2 - very or somewhat dissatisfied	3 (7.50%)	1 (10.00%)	0 (0.00%)	1 (10.00%)	1 (10.00%)	
3 or 4 - very or somewhat satisfied	37 (92.50%)	9 (90.00%)	10 (100.00%)	9 (90.00%)	9 (90.00%)	
**In your expert opinion, would the chatbot's response pose harm if provided to an actual patient?**						
No	40 (100.00%)	10 (100.00%)	10 (100.00%)	10 (100.00%)	10 (100.00%)	

**Variable**	**Overall**, N = 40[1]	**Ant. Segment/ Glaucoma**, N = 10[1]	**Neuro-ophth**, N = 10[1]	**Ped/ Oculo -plastics**, N = 10[1]	**Retina**, N = 10[1]	**p-value**[2]

[1] n (%)

[2] Fisher's exact test

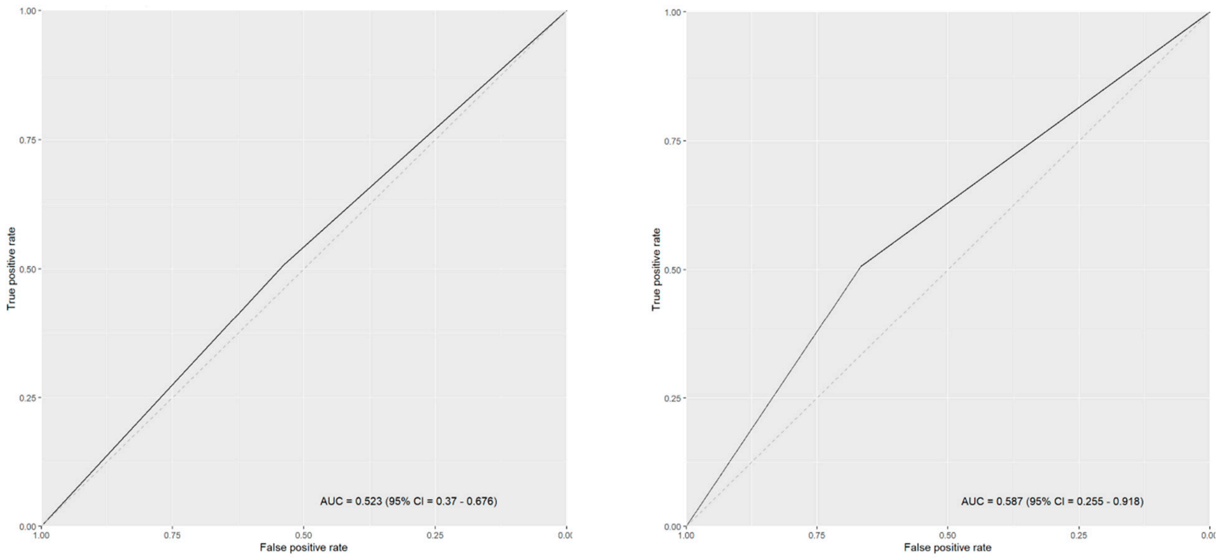

A B

Figure S1. Receiver operating characteristic curves for Bard (A) and GPT-4 (B) in providing triage recommendations. The model performance of the degree of detail in prompt predicting the appropriate triage for ChatGPT (AUC 0.587) is better than Bard (AUC 0.523).
